# Design of Catalytically Amplified Sensors for Small Molecules

**DOI:** 10.3390/biom4020402

**Published:** 2014-04-17

**Authors:** Olga V. Makhlynets, Ivan V. Korendovych

**Affiliations:** Department of Chemistry, Syracuse University, Syracuse, NY 13244, USA

**Keywords:** sensor, protein design, signal amplification, catalysis

## Abstract

Catalytically amplified sensors link an allosteric analyte binding site with a reactive site to catalytically convert substrate into colored or fluorescent product that can be easily measured. Such an arrangement greatly improves a sensor’s detection limit as illustrated by successful application of ELISA-based approaches. The ability to engineer synthetic catalytic sites into non-enzymatic proteins expands the repertoire of analytes as well as readout reactions. Here we review recent examples of small molecule sensors based on allosterically controlled enzymes and organometallic catalysts. The focus of this paper is on biocompatible, switchable enzymes regulated by small molecules to track analytes both *in vivo* and in the environment.

## 1. Introduction

**Figure 1:**
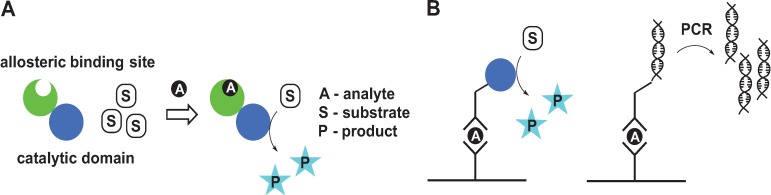
The general principle of analyte detection by catalytically amplified sensor (**A**) and comparison of ELISA and IPCR (**B**).

Small molecule sensors have a broad range of applications ranging from metal tracking *in vivo* to detecting chemicals in food and the environment. One common strategy for sensor design utilizes conformational switches that change their structure upon analyte binding, resulting in a change in fluorescence of reporter groups [1,2,3,4,5,6,7,8,9,10,11]. Another approach, which is the focus of this review, is based on catalytically amplified sensing in which allosteric [12] binding of an analyte triggers conformational changes, which in turn modulates catalysis at the active site (Figure 1A). This method effectively links a *single* analyte binding event with production of *multiple* molecules of colored and/or fluorescent product that can be easily measured. Owing to the large signal amplification, catalytically amplified sensing provides a significant sensitivity advantage over sensors that rely on fluorescence turn-on due to change in relative orientation of reporter groups. Some of the best-known examples of catalytically amplified sensors are enzyme-linked immunosorbent assay (ELISA) [13,14,15] and immuno-polymerase chain reaction (IPCR) [16,17]. ELISA is a plate-based assay designed to identify and quantify biological analytes, including proteins, peptides and hormones. While ELISA assays are not purely allosteric, they demonstrate nicely the concept of signal amplification through catalysis (Figure 1B). An antibody immobilized on a solid support selectively interacts with antigen in the liquid sample. After removal of unbound antigen the plate is treated with antigen-specific antibody conjugated to an enzyme that produces a colored or luminescent product. A variety of enzymes can be used to amplify the signal, but the most common enzyme is horseradish peroxidase (HRP) in combination with 3,3',5,5'-tetramethylbenzidine (TMB) or 2,2'-azino-bis(3-ethylbenzothiazoline-6-sulfonic acid) (ABTS), which produce colored product in the presence of H_2_O_2_. IPCR combines the versatility of the ELISA approach with the sensitivity of PCR. In IPCR, an antibody is linked to DNA instead of the enzyme (Figure 1B) and the signal is amplified exponentially using a polymerase chain reaction [18,19,20]. DNA amplicons generated by PCR can be quantified by gel electrophoresis or real-time fluorescence measurements [20,21] improving detection limit by 100–100,000-fold as compared to ELISA.

Several recent reviews [22,23] have already discussed key quantitative principles of analyte detection through signal amplification, and here we provide only a brief summary. When designing sensors one must consider such properties as detection limit, linear relationship between the analyte concentration and signal output, and the response time. For an efficient sensor the following conditions need to be met: (1) the rate of analyte binding by sensor should be faster than the rate of the reporter reaction; (2) sensor concentration should be sufficient to bind all analyte molecules; (3) low background rate when analyte is not bound; (4) extinction coefficient or quantum yield of the product should be as high as possible. In an ideal situation all analyte (A) is bound to the sensor and the readout signal (product concentration P) is amplified by a factor k_cat_*∆t, where k_cat_ represents the catalyst’s turnover number and ∆t—reaction time: [P] = k_cat_*∆t [A]. High k_cat_, high affinity of the sensor for substrate, and high extinction coefficient for colorimetric output signal or quantum yield for fluorometric output signal are essential parameters for signal amplification.

Much attention has been devoted to catalytically amplified sensors for detection of proteins and DNA [24,25,26,27,28,29,30]. In this review we will highlight recent examples of protein-based sensors for small molecules and compare them to organometallic catalysts that switch on upon analyte binding. Protein-based sensors offer several critical advantages for sensor design. First, nature has already created a multitude of proteins that recognize various ligands and catalyze many reactions with high efficiency, providing an excellent starting point for developing new sensors. Second, modern molecular biology techniques allow for easy expression and purification of large quantities of various proteins. Finally, new proteins can be designed using directed evolution to bind previously unrecognized ligands or to catalyze unnatural reactions. The field of biosensor design holds much promise for environmental control, explosives detection and therapeutic applications such as sensing disease markers, and measuring concentrations of metal ions, hormones, *etc*. In addition to its inherent practical value, biosensor design research advances fundamental knowledge of protein structure and function.

Many of the designs that take advantage of existing allosteric regulations have been previously reviewed [11,31,32,33,34,35,36,37]. Here we survey progress in sensor design as well as challenges of engineering novel binding and/or catalytic sites in non-enzymatic proteins.

## 2. Signal Amplification by Protein-Based Switches

Here we review recent examples of protein-based sensors implementing catalytic signal enhancement. Each subsection represents a different design strategy.

### 2.1. Control of Enzyme Assembly

**Figure 2:**
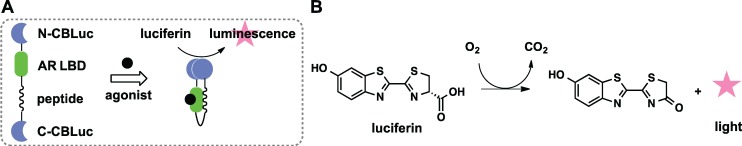
Bioluminescent sensor for agonist detection based on complementation strategy of split CBLuc. (**A**) Luciferase was split in two halves that reassemble when agonist is present. The enzyme domain is in blue, the allosteric binding domain is in green, and the signal that modulates the switch is shown as a black sphere; (**B**) Oxidation of luciferin promoted by luciferase generates bioluminescent light.

Correct fold of enzyme is essential for its activity, therefore controlling the protein’s structure is a way to modify its function. Kim *et al.* used this strategy to build a probe for detection of androgen receptor (AR) agonists [38,39]. Click beetle luciferase (CBLuc) was split in two parts, giving functionally inactive fragments. The ligand-binding domain of androgen receptor (AR LBD) was fused to a coactivator peptide and the resulting fusion construct was placed between the *C*-terminal and *N*-terminal parts of CBLuc (Figure 2A). Androgen receptor agonist dihydroxytestosterone (DHT) induces association between AR LBD and the peptide, bringing together the *N*- and the *C*-terminal fragments of CBLuc thus restoring its activity (Figure 2B). CBLuc is insensitive to pH and metal ions and therefore provides an excellent *in vivo* probe. Live human cells expressing the probe produced tissue-transparent red bioluminescence signal ~30 times higher than a control upon exposure to 10 μM DHT for 20 min. After washing cells to remove DHT, the luminescence intensity decreased to background levels within 2 h [38]. The probe was shown to be agonist-selective and may have applications in screening for chemicals that induce AR signaling. In another study the same group developed a combination of sensors that emit characteristic red or green bioluminescence light in response to estrogen receptor agonist or antagonist, respectively [39].

### 2.2. Domain Insertion

**Figure 3:**
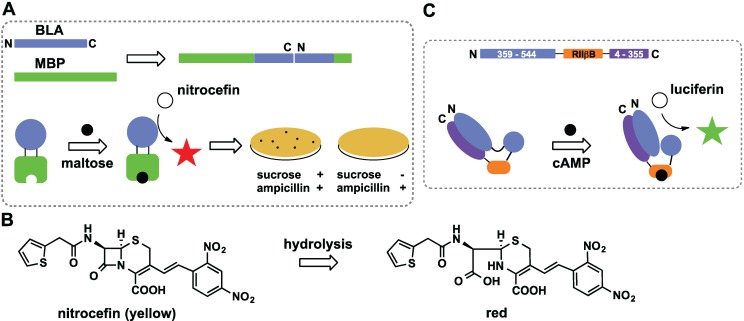
Insertion of one protein into another using circular permutation. (**A**) β-lactamase (BLA) was first permuted and then inserted into maltose binding protein (MBP), binding of maltose induces conformational changes that trigger hydrolysis of nitrocefin into a red product. Plating cells expressing the switch on β-lactam antibiotics allows engineering of probes responsive to ligands other that maltose; (**B**) Nitrocefin hydrolysis catalyzed by β-lactamase produces a red product; (**C**) Firefly luciferase engineered to detect cAMP. Binding of analyte to RIIβB promotes conformational change that increases bioluminescence.

Domain insertion creates a chimera that couples the ligand binding capability of one protein to the catalytic activity of another (this method is reviewed in detail in [37]). Usually, the guest protein is inserted into a loop of the host protein to avoid structure perturbation. The Ostermeier group has combined maltose binding protein (MBP) and β-lactamase (BLA) to create a protein switch whose catalytic activity is regulated by maltose [40,41]. They used circular permutation to change the topology of BLA by joining the original *C*- and *N*-termini with a linker and cutting the sequence at a different site (Figure 3A). This rearrangement allowed for coupling of two unrelated proteins in order to create a catalytically amplified sensor. Screening of a library of chimeras with different permutation sites identified a switch with β-lactamase ability ~600-fold higher in the presence of maltose as compared to the background. The activity was monitored using nitrocefin (Figure 3B), a β-lactam that produces a colored product upon hydrolysis. Expression of the fusion protein in *E. coli* provides maltose-dependent β-lactam antibiotic resistance to the cells. This phenotype was used to create new sensors that responded to different ligands. The maltose-binding site was altered and proteins that responded to sucrose binding were selected by plating cells on ampicillin plates in the presence of sucrose (Figure 3A). Colonies from sucrose-containing plates were further screened for nitrocefin hydrolysis and one of the switches had high affinity for sucrose (0.7 μM) and relatively high specificity (30-fold higher for sucrose as compared to maltose). In other studies by the Jones lab, the heme binding function of cytochrome b was coupled to antibiotic degrading activity of β-lactamase to create a switch responsive to heme [42,43].

A sensor for detection of cAMP was developed by circular permutation of firefly luciferase [44]. Crystal structure of luciferase reveals that the enzyme consists of two domains connected by a hinge. Upon substrate binding the two domains rotate and assume closed conformation. The protein was subjected to circular permutation creating an inactive enzyme, where the domains cannot rotate freely around the hinge (Figure 3C). A cAMP-binding domain B from kinase regulatory subunit type IIβ (RIIβB) was then fused between native *N* and *C* termini of luciferase through a set of linkers. Optimization of permutation site and linker size produced a construct with 70-fold increased luciferase activity in the presence of 100 μM cAMP as compared to the background.

### 2.3. Protein Splicing

**Figure 4:**
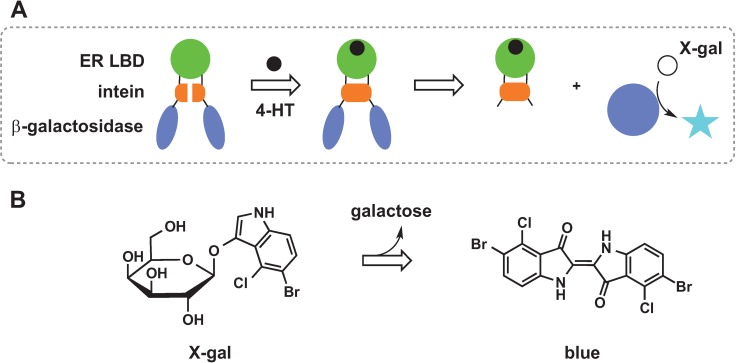
Sensor design based on protein splicing. Ligand binding domain (ER LBD), intein, and β-galactosidase enzyme are fused together so that binding of the ligand activates splicing of the enzyme (**A**), which then catalyses X-gal hydrolysis and formation of a blue product (**B**).

An intein is a part of a protein that can cut itself out, joining the remaining protein sequences by a peptide bond in a process called protein splicing. Two groups engineered sensors by insertion of a hormone binding domain into an intein, which was then inserted into a reporter protein (enzyme). Insertion of a sequence into a splicing domain abolishes splicing ability, which is then restored upon binding of the hormone. The subsequent protein splicing removes intein and joins two halves of the enzyme, activating its function. Buskirk *et al.* used estrogen receptor ligand binding domain (ER LBD) that undergoes a conformational shift upon binding of 4-hydroxytamoxifen (4-HT) placing the *N*- and *C*-termini closer to each other [45]. This protein was inserted into *Mycobacterium tuberculosis* RecA intein, which in turn was inserted into β-galactosidase (Figure 4A). The β-galactosidase activity was measured by hydrolysis of X-gal, which produces a blue product (Figure 4B). Significant X-gal hydrolysis was measured only in the presence of 4-HT. The splicing ability upon 4-HT binding was also linked to a red colony phenotype in *Saccharomyces cerevisiae*. Ade2 enzyme is involved in the adenine biosynthesis pathway and its absence results in accumulation of a bright red byproduct. Insertion of ER-intein fusion into the gene encoding Ade2 abolished Ade2 activity as shown by formation of red yeast colonies. However, addition of 4-HT triggered Ade2 splicing, restoring enzyme activity and producing normal white cells. This sensor can serve as a tool to control protein function in living cells via a small cell-permeable molecule. Skretas *et al.* designed a probe whose splicing ability and thus β-galactosidase (β-lactamase) activity were controlled *in vivo* by the presence of a thyroid hormone [46]. Random mutagenesis combined with genetic selection through growth phenotype produced intein that could be controlled by the presence of synthetic estrogen ligands in a dose-dependent fashion.

### 2.4. Chemical Rescue

**Figure 5:**
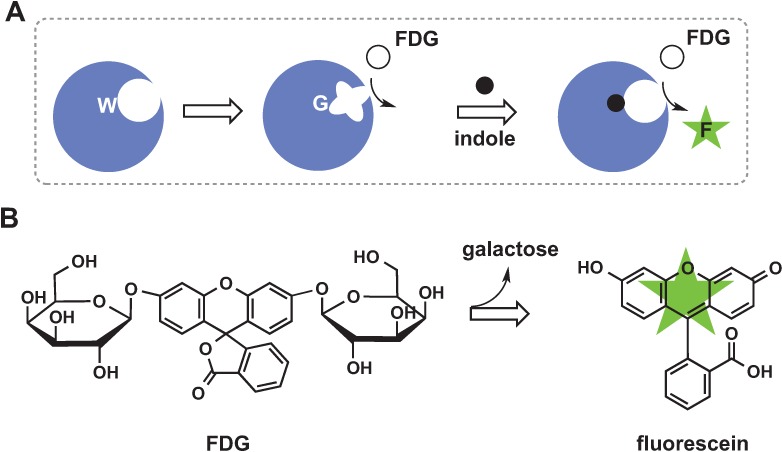
Protein sensor designed using chemical rescue approach. (**A**) A single mutation changes the structure of the active site, rendering the enzyme inactive; (**B**) Addition of indole restores the structure and rescues the enzyme’s ability to catalyze FDG hydrolysis to the wild type level.

This method is based on introducing a mutation that disrupts structural integrity of the active site and leads to inactive enzyme. Addition of a small molecule restores structure and function of the enzyme. Deckert *et al.* designed a switch to modulate β-glycosidase activity by indole [47,48] (Figure 5A). A single tryptophan located close to the active site was mutated to a glycine and the resulting protein had ~700-fold lower k_cat_/K_M_ as compared to wild type enzyme. The crystal structure of this mutant showed that a key residue at the active site shifted to fill the cavity formed by the Trp to Gly mutation and thus moved away from the substrate. The added indole occupied the cavity and restored the enzyme’s structure and function (as measured by hydrolysis of fluorescein *di*-β-d-galactopyranoside, FDG, Figure 5B). In this example the mutation was introduced very close to the active site, however the strategy described may be used for introducing mutations at a remote site given that disruption of the structure is relayed to the active site.

Lin *et al.* reprogrammed zinc finger transcription factor C7 to respond to small-molecule binding and turn on luciferase gene transcription [49]. The two critical residues (His125 and Phe116), which are responsible for zinc binding and hydrophobic packing, were mutated to glycine and alanine, respectively. These mutations significantly perturbed the integrity of DNA binding helix. A library of 250 small molecules was screened to identify molecules that complement mutations and rescue DNA binding function. One of the most successful candidates, 2-(4'-quinoline)benzimidazole induced luciferase activity by 18-fold. Further studies by surface plasmon resonance demonstrated that interaction of this compound with the mutant zinc finger increased affinity of the mutant C7 to its cognate DNA sequence.

### 2.5. De Novo Design of Catalytic Function

**Figure 6:**
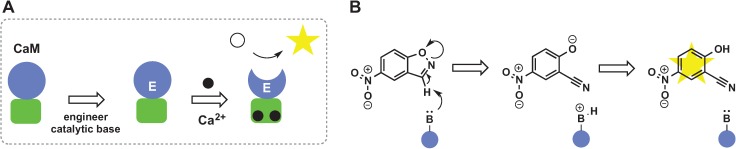
The general principle of Ca^2+^ detection using calmodulin (CaM) engineered to catalyze Kemp elimination. (**A**) CaM undergoes structural changes upon Ca^2+^ binding that lead to formation of a hydrophobic pocket. Introduction of catalytic residue Glu92 into this pocket enabled calmodulin to catalyze the Kemp elimination reaction (**B**).

Calmodulin (CaM) is one of the most abundant eukaryotic regulatory proteins involved in many cellular processes. CaM consists of two independent helical domains connected by a linker. Each of the two domains has two EF-hand motifs. Calcium binding by EF-hand triggers a drastic conformational change resulting in formation of solvent exposed hydrophobic cavities in each of its domains. This unique ability was previously exploited to create several successful fluorescent switches, including pericams [3], cameleons [2] and camgaroos [4]. However, those sensors are not catalytically amplified as CaM has no enzymatic activity of its own. Korendovych *et al.* used computational protein design to introduce catalytic activity into this non-enzymatic scaffold. The *C*-terminal domain of CaM was chosen as a scaffold because of its naturally high affinity for Ca(II), high thermodynamic stability, presence of a cavity that can accommodate substrate, and its small size to facilitate preparation and NMR studies of the sensor. A single Glu residue introduced at the bottom of an otherwise hydrophobic pocket confers the ability to catalyze Kemp elimination on CaM and converts it into a catalytically amplified sensor for Ca(II), named AlleyCat [50] (Figure 6A). Kemp elimination is an excellent choice for a readout reaction as substrate is easily available and concentration of product can be measured spectrophotometrically (Figure 6B). In addition, there are no natural enzymes in *E. coli* that catalyze this reaction and therefore catalytic activity can be measured in crude cell lysates, a factor important for directed evolution. The catalytic efficiency of the original AlleyCat design was improved over 200-fold after 7 rounds of directed evolution [51]. Thus AlleyCat is an efficient catalytically amplified sensor for calcium that could be used both *in vivo* and *in vitro*. The key advantages of using the *de novo* protein design to create catalytically amplified sensors are: (1) catalytic reaction of choice can be used to amplify the signal; (2) both binding site and catalytic site can be evolved to improve selectivity and catalytic efficiency. To show that this approach can be extended to other metals, Mack *et al.* redesigned the EF-hand motif to sense trivalent metal ions by mutating two neutral residues in the Ca^2+^-binding loop to glutamates. The resulting protein (CuSeCat) has almost two orders of magnitude higher selectivity for trivalent metals as compared to calcium [52].

### 2.6. Switches Based on Two-Component Systems

**Figure 7:**
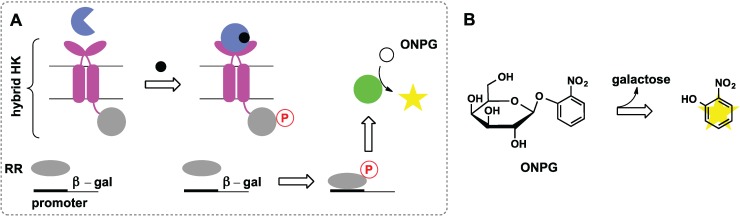
Synthetic two-component pathway that combines redesigned receptor (blue) with chimeric histidine kinase (HK) consisting of periplasmic domain from chemotaxis sensor (pink) and cytoplasmic domain from osmosensor (grey). (**A**) This chimeric HK phosphorylates response regulator (RR, grey) from osmosensor, which then turns on transcription of the β-galactosidase gene (green); (**B**) Reaction used to measure β-galactosidase activity. ONPG, 2-nitrophenyl-β-galactopyranoside.

Two-component signal transduction systems (TCSs) consist of histidine kinase (HK) and cytosolic response regulator (RR). Analyte binding at the extracellular part of HK results in conformational changes that send the signal across the membrane and trigger phosphorylation of histidine at the intracellular tail. An aspartate on the response regulator is then phosphorylated, which induces a response by up-regulating gene expression. In wild type pathways, periplasmic ribose-binding protein (RBP) binds ribose, which initiates a signal cascade that controls chemotaxis. Several studies from the Hellinga group showed that RBP can be redesigned to bind Zn(II) [53], trinitrotoluene, and l-lactate [54], and these receptors were introduced into TCS that regulates β-galactosidase gene (Figure 7A). The resulting synthetic TCSs were shown to regulate gene expression in response to extracellular ligands as measured by increased β-galactosidase activity (Figure 7B). However, it should be noted that these studies were later questioned as no binding of the designed receptors to their respective ligands could be detected experimentally [55].

More recently, *Salmonella* iron(III)-sensing TCS was modified to respond to lanthanide ions. The iron(III)-binding motif was replaced with a lanthanide binding sequence and the resulting hybrid TCS was shown to regulate expression of GFP fused to the corresponding promoter [56]. This work paves the way to engineered organisms for detection of metal ions in the environment.

While the TCS system approach relies on enzyme expression as a function of analyte concentration, it is possible to report the change in substrate concentration when enzyme concentration remains constant. Specifically, hydrolysis of exogenously added nitrocefin by periplasmic β-lactamase or hydrolysis of ONPG by cytoplasmic β-galactosidase was used to measure permeability of outer and inner membranes, respectively [57,58,59]. This strategy therefore may be used to sense small molecules that are capable of permeabilizing the membrane.

## 3. Signal Amplification by Organometallic Catalysts

In the following section we summarize recent successful reports on design of allosterically controlled organometallic catalysts with convenient colorimetric or fluorometric readouts supplementing extensive in-depth reviews on this subject [23,60,61,62,63].

### 3.1. Switches Based on a Weak-Link Approach

**Figure 8:**
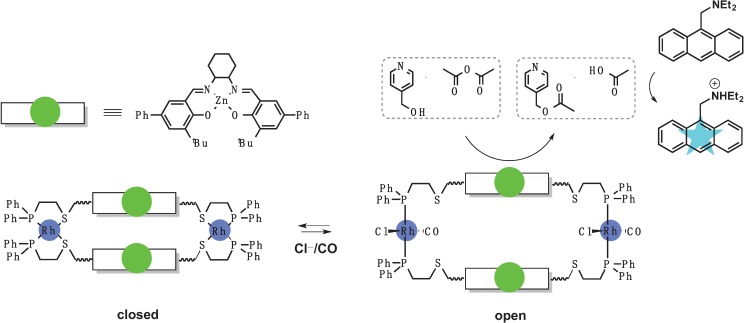
Allosterically controlled macrocycle catalyst designed through WLA. Recognition center is marked blue and catalytic center is in green. CO and Cl^−^ binding breaks weak-link Rh-S bonds and converts structure into a catalytically active “open” state.

Using a weak-link approach [64,65] Gianneschi *et al.* created a supramolecular reversible allosteric detection system with catalytically amplified signal that can be activated by small molecules such as Cl^−^ and CO [66]. The recognition part of the sensor is comprised of metal ions (Rh(I) or Cu(I)) bound to flexible hemilabile ligands with sulfur and phosphorus as the donor atoms (Figure 8). The flexible portion of the ligand, which forms the catalytic part of the sensor, contains a Zn-Salen complex that is not sterically accessible in the absence of Cl^−^ and CO. When Cl^−^ or CO bind to the sensor, they displace the thioether sulfurs in the Rh(I) coordination sphere, while keeping stronger Rh-P bonds intact. The resulting coordination sphere rearrangement leads to an extension of the macrocycle cavity (shown as the open state in Figure 8) and subsequent exposure of the catalytic component of the sensor. The rearrangement turns on the catalysis of a transfer reaction between acetic anhydride and pyridyl carbinol. The rate of the reaction is 25-fold higher relative to the background rate in the absence of Cl^−^ and CO. The readout is simplified by the presence of a pH-sensitive fluorophore, which reports how much acetic acid was generated. Reversible switching of the sensor to the closed inactive state was achieved by purging the system with nitrogen or removing CO under vacuum.

In another proof-of-concept study, Yoon *et al.* used a Zn-Salen based system, very similar to the one described above, for acetate detection with catalytic signal self-amplification [67]. Acetate binding to the Rh(I) center opens the macrocycle, initiating a catalytic reaction between acetic anhydride and pyridyl carbinol to produce more acetic acid. A pH-sensitive fluorophore-base then reacts with acetic acid to form acetate, which in turn self-amplifies the reaction. The system demonstrates that organometallic probes can be designed to amplify the signal via a cascade of additional catalytic cycles, similarly to polymerase chain reaction cascade. Furthermore, it was shown that phenanthroline can open the complex when Rh(I) is replaced with Cu(I) and therefore the design could be extended to detection of phenanthroline [68]. Using the same strategy another complex was designed that could reversibly operate in pseudo-aqueous conditions with good catalytic rate and turnover. Instead of the Zn-Salen motif, the macrocycle featured a Zn-pyridine-bisimine core with two zinc centers bridged by an acetate ion, which prevented substrate access to the catalyst [69]. As expected, no catalytic hydrolysis of 2-(hydroxypropyl)-*p*-nitrophenyl phosphate (HPNP) was observed in the closed state, however CO/Cl^−^ binding opened the macrocycle and increased the rate 100-fold as measured by formation of colored *p*-nitrophenolate.

### 3.2. Metal Sensors Based on Variable Affinity to Ligands

**Figure 9:**
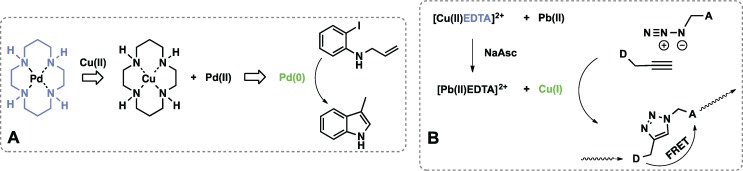
Sensors for metals designed based on differences in ligand affinity. (**A**) Cu(II) is recognized by cyclam ligand (blue), release of Pd(II) and its *in situ* reduction by phosphine to catalytic Pd(0) (green) enables the Heck reaction; (**B**) Free Cu(II) displaced upon Pb(II) binding is reduced to Cu(I) which catalyzes formation of the FRET active product through Huisgen cycloaddition.

Two catalytically amplified sensors for Cu(II) and Cd(II) were designed in the Anslyn lab based on Heck reaction [70,71]. The sensor for copper is based on the competition between Cu(II) and Pd(II) for polyaza cyclam ligand (PAC). When complexed with PAC, Pd(II) can not catalyze formation of fluorescent 3-methylindole via the Heck reaction. Added Cu(II) releases a certain amount of Pd(II), which after phosphine-mediated reduction to Pd(0) catalyzes formation of 3-methylindole (Figure 9A). Selectivity for Cu(II) is driven by high affinity of PAC ligand for this metal ion, as compared to other metal ions. A very similar strategy was applied to a design of Cd(II) sensor. A ligand selective for Cd(II), 1,4,7,10,13-pentaazacyclopentadecane, inactivated catalytic formation of fluorescent 7-diethylaminocoumarin. Cd(II) sequestered the ligand releasing Pd(II), which was then reduced *in situ* and catalyzed the fluorogenic Heck reaction.

In another study, azide alkyne Huisgen cycloaddition catalyzed by Cu(I) was used as a platform to report presence of Pb(II) ions [72]. EDTA coordinates Cu(II) and prevents its reduction to catalytically active Cu(I). Addition of Pb(II) releases Cu(II), which after reaction with ascorbate catalyzes a cycloaddition reaction between azide and alkyne attached to fluorophores (Figure 9B). Förster resonance energy transfer (FRET) between anthracene (donor, D) and coumarin (acceptor, A) correlates with the amount of product formed and reports the concentration of the analyte.

### 3.3. Sensors Based on Auto Amplification Mechanism

**Figure 10:**
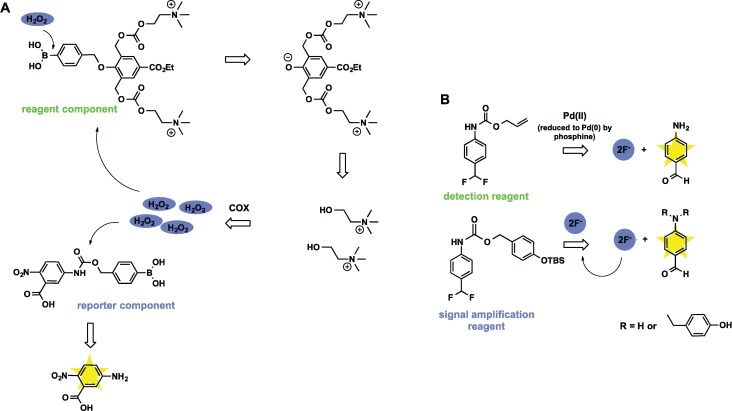
Sensors for H_2_O_2_ and Pd(II) that utilize autocatalytic signal amplification. (**A**) A molecule of H_2_O_2_ triggers cleavage and disassembly of reagent component, the process which in combination with COX catalysis produces many more H_2_O_2_ and initiates a dendritic chain reaction; (**B**) Pd(II) reacts with detection reagent with release of F^−^, which in turn reacts with amplification reagent and produces more F^−^ in an autocatalytic fashion.

Sensors for H_2_O_2_ [73] and fluoride [74] were designed in the Shabat group using decomposition of dendrimers through a chain reaction. In this approach the analyte removes a triggering group, which in turn produces more analyte and initiates additional reaction cycles and ultimately results in exponential growth of the signal generated by a reporter molecule. Two components were used to achieve signal amplification for hydrogen peroxide detection (Figure 10A). A chain reaction is initiated when a H_2_O_2_ molecule cleaves phenylboronic acid (trigger) of the reagent component and releases two choline molecules. Choline oxidase (COX) present in the solution oxidizes each choline molecule with formation of two H_2_O_2_ molecules. When H_2_O_2_ removes phenylboronic acid group from a reporter component, yellow 5-amino-2-nitrobenzoic acid is released and can be detected by spectrophotometer.

A similar approach has been applied by Baker *et al.* who developed an autocatalytic system for palladium detection that also requires two components for detection and amplification [75]. The detection reagent reacts with palladium to release fluoride, which then triggers an autocatalytic reaction with the signal amplification reagent producing a product (Figure 10B).

## 4. Conclusions

The field of sensor design has grown significantly over the past decade due to interest in the detection of disease biomarkers, metal homeostasis, environmental pollutants and explosives. Although many sensitive methods for small molecule detection already exist (e.g., ICP, ITC, SPR), they often require sophisticated instrumentation and timely sample preparation. Catalytically amplified sensors provide advantages of quick signal readout based on formation of colored or fluorescent product that can be clearly observed visually and/or quantitatively measured by simple spectrophotomer/fluorometer. These qualities are indispensable when working at remote sites or without access to instrumentation.

Here we have reviewed a number of successful approaches for the design of catalytically amplified sensors. While promising results have been achieved in the field of organometallic sensors, their relatively low selectivity, high background reaction rates and narrow solvent compatibility limit their practical applicability. On the other hand, protein-based sensors are more robust and can be optimized by directed evolution. However, current state-of-art protein-based catalytically amplified sensors still mostly utilize natural machinery and existing allosteric interactions. Recent developments in *de novo* protein design give hope that this weakness will soon be overcome, providing essentially limitless abilities for cheap, efficient and biocompatible sensors for small molecules.
